# Bending Behavior of Fiber Metal Laminate Plates Under Thermo-Mechanical Loads

**DOI:** 10.3390/ma18194640

**Published:** 2025-10-09

**Authors:** Like Pan, Tong Xing, Yingxin Zhao, Yuan Yuan, Caizhi Yang

**Affiliations:** Standards & Metrology Research Institute, China Academy of Railway Sciences Corporation Limited, Beijing 100015, China; plk1986@126.com (L.P.); yyzj2003@163.com (Y.Y.); 15201227298@163.com (C.Y.)

**Keywords:** fiber metal laminate plates, thermo-mechanical loads, orthotropy, state space method, temperature dependence

## Abstract

An exact analytical model based on three-dimensional (3D) thermo-elasticity theory is developed to investigate the bending behavior of fiber metal laminate (FML) plates under thermo-mechanical load. The temperature-dependent properties and the orthotropy of the component materials are considered in this model. The analytical model is based on the heat conduction theory and thermoelasticity theory, and the solutions are determined by employing the Fourier series expansion, the state space approach and the transfer matrix method. Comparison study shows that the FE results are generally in good agreement with the present analytical solutions, exhibiting relative errors of less than 2%, except in the regions near the upper and lower surfaces. The present solution is close to the experimental values for the laminated plate within the linear range, with errors less than 10%. The decoupling analysis indicates that the thermo-mechanical performance of FML plates no longer strictly adheres to the traditional superposition principle, with errors reaching 30.39%. A modified principle accounting for modulus degradation is introduced to address this discrepancy. Furthermore, parametric studies reveal that the temperature and the lamina number have significant effect on the stresses and displacements of the FML plate.

## 1. Introduction

Fiber metal laminates (FMLs) are a class of multi-phase layered laminates that combine the ductility of metals with the high specific strength and stiffness of fiber-reinforced polymer (FRP) [1,2]. Owing to their excellent bending performance, design flexibility, impact resistance, fatigue resistance, and other favorable properties, FMLs have found widespread applications in aerospace, railway transportation, and civil engineering, such as bridge deck surfacing, bridge reinforcement, and aircraft skin structures. Typical constituent materials used in FML structures include metals such as aluminum (Al), magnesium (Mg) and titanium (Ti) alloys, as well as fibers commonly found in FRPs, such as glass fiber, carbon fiber, and aramid fiber. In practical engineering applications, FML plates often serve as bending members and are subjected to complex thermo-mechanical service environments, such as aerodynamic heating during high-speed flight and stress concentrations caused by thermal expansion under high-temperature conditions. The temperature not only affects the stress distribution within the structure but also leads to material property degradation, thereby reducing the overall capacity of the structure. Therefore, the bending response of FML plates in thermo-mechanical loads is worth studying for enhancing structural safety.

Experimental investigation is one of the primary approaches to studying the mechanical behavior of FML plates. Over the past several decades, extensive research has been devoted to understanding the bending behavior of FMLs and the influence of temperature. The influence of thermal cycling on the bending strength and other mechanical performance of advanced FMLs incorporating Al–lithium alloy was comprehensively examined by Li et al. [3]. Wang and Anderson [4] investigated the bending response of FMLs with various stacking sequences subjected to repeated cryogenic cycles, where each cycle lasted for 4 min between −100 °C and 25 °C. The properties of FMLs can be enhanced by mixing nano-scale particles. Based on different volume fractions of the additive, Li et al. [5] investigated the effect of graphene oxide on the bending and other mechanical properties of FMLs. For different aerospace applications, novel FMLs were developed by Hussain et al. [6] using nickel-Ti with carbon fiber and investigated the effect of various temperatures on the bending properties of these novel FMLs. Hu et al. [7] investigated the bending and mechanical properties of FMLs with various layup configurations and fiber orientations, including 0°, 90°, and ±45°. To enhance FMLs’ bending and other mechanical characteristics, Logesh and Bupesh Raja [8] added various contents of Mg–Al-layered double hydroxides into FMLs, and the resulting laminates were studied through a series of experiments. Vasudevan et al. [9] developed copper-based FMLs via hand layup, incorporating carbon nanotubes and varied stacking sequences, and conducted extensive mechanical tests to enhance bending, tensile, fatigue, and wear performance. Hu et al. [10] investigated the effects of temperature variation on the mechanical behaviors of polyetheretherketone-based FMLs to evaluate their temperature resistance capability. The effect of the assembly method between composite prepreg and Al sheets as well as the stacking configurations on the bending response of FMLs were investigated by Sang et al. [11]. The synergistic effects of temperature and loading speed on the laminate deformation under bending and tensile conditions were investigated and analyzed by Jin et al. [12].

Experimental investigations have played a crucial role in advancing the understanding of the mechanical behavior of FMLs, offering direct and reliable data that closely reflect real-world conditions. Such methods also involve considerable time, cost, and complexity, particularly when dealing with a wide range of loading and environmental scenarios. Analytical approaches based on rigorous theoretical models provide a valuable complement. They not only help uncover the underlying physical mechanisms that govern the structural response but also offer greater flexibility, efficiency, and cost-effectiveness in studying diverse conditions. Jin et al. [13] developed a 3D constitutive model to analyze the mechanical behavior of fiber metal laminates under both bending and tensile conditions with improved prediction accuracy. Abouhamzeh et al. [14] proposed a general thermo-viscoelastic framework capable of predicting thermal stress and deformation in composites with arbitrary geometries and orthotropic properties. A higher-order shear deformable theory for laminated composite plates was proposed by Aydogdu [15], derived from three-dimensional elasticity bending solutions through an inverse method. Ghomshei et al. [16] employed the differential quadrature method to analyze the thermal buckling behavior of symmetric cross-ply laminated rectangular thin plates subjected to both uniform and non-uniform temperature fields. Chen et al. [17] proposed a bending model for CFRP-Al laminates by integrating modified laminate theory with metal plasticity and interface interaction. A novel displacement-based higher-order theory was developed by Ali et al. [18] for the analysis of structures under combined mechanical and thermal loading conditions. A variationally consistent third-order shear deformation theory for laminated plates, which ensures transverse shear stress continuity at interlaminar interfaces, was developed by Wang and Shi [19]. Based on the refined plate theory, Jiang et al. [20] presented an analytical solution for the three-dimensional thermodynamic analysis of a piezoelectric laminated plate subjected to a steady three-dimensional temperature field. Han et al. [21] proposed an efficient and accurate thermal stress analysis method based on the conventional first-order shear deformation theory. The Fourier heat conduction equation was solved by Brischetto and Carrera [22] for layered structures incorporating orthotropic layers in order to obtain the temperature distribution along the thickness direction. To study the degradation of FML fire performance, Grigoriou and Mouritz [23] developed a coupled thermo-mechanical model capable of predicting tensile and buckling strength reduction during fire exposure. Similarly, Feih et al. [24] estimated failure times of polymer laminates under unidirectional flame radiation via a coupled thermal-mechanical failure model. Asaro et al. [25] proposed a time-dependent failure model to explain the damage evolution in FRP composites under thermo-mechanical loading. In existing studies, most analytical models are based on the transverse shear deformation assumption, such as the Kirchhoff plate theory, first-order and higher-order shear deformation theories, which are applicable to thin and moderately thick plates, but the errors increase with plate thickness. Moreover, the influence of temperature on material properties is often neglected, and limited attention has been given to the bending response of FML plates under coupled thermo-mechanical loading conditions.

This paper presents a Fourier series-based method for analyzing a three-dimensional elastic layered composite, fully incorporating transverse shear deformation effects, for the bending behavior of FML plate under thermo-mechanical load. In addition, the orthotropic mechanical property of FRP layers and the temperature-dependent material properties are both taken into account by the present model. By employing Fourier series expansion, the three-dimensional governing equations for temperature, stress, and displacement are transformed into one-dimensional ones. The state-space approach, coupled with the transfer matrix method, is utilized to enforce interlayer continuity, allowing for the analytical determination of field variables under specified surface temperature and loading conditions. Finally, the accuracy of the analytical model is evaluated through comparison with finite element results. In addition, the decoupling analysis and the parametric studies on the bending behavior of FML plate under thermo-mechanical loads are conducted.

## 2. Thermo-Mechanical Analytical Model of FML Plate

**Figure 1:**
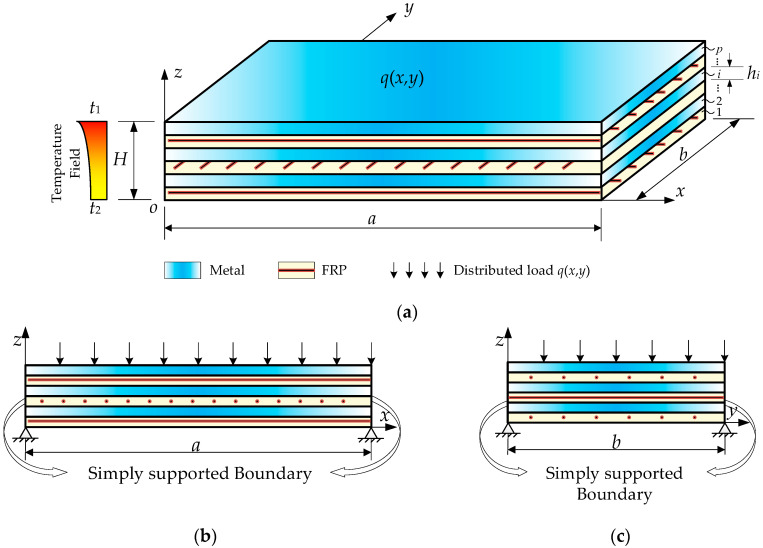
Schematic diagram for FML plate under thermo-mechanical loads. (**a**) Perspective view; (**b**) front view; (**c**) side view.

The structural member illustrated in Figure 1 is a FML plate that comprises *p*-stacked FRP and metallic laminae, each having a thickness *h_i_* (*i* = 1, 2, …, *p*). The thickness, material, and stacking sequence of each lamina can be arbitrary. The length, width, and thickness of the FML plate are, respectively, *a*, *b*, and *H*, in which $H=\sum_{i=1}^{p}h_{i}$. The adjacent laminae are well-bonded without interfacial slip. The plate is simply supported along all edges, subjected to a distributed surface load *q*(*x*, *y*) applied on the top face, and exposed to a non-uniform temperature field, with prescribed temperatures *T*_1_ and *T*_2_ on the upper and lower surfaces, respectively. At elevated temperatures, the modulus of both FRP and metallic laminae degrades, with $\phi_{i}$ denoting the modulus degradation factor induced by temperature of the *i*-th lamina.

### 2.1. Basic Assumptions and Applicability Scope

(1) There is no internal heat generation, and that convective and radiative heat losses are neglected;
(2) The temperature analysis considers only steady-state heat conduction;
(3) The FML plate deforms within the linear range;
(4) The adjacent laminae are well-bonded, and the interfacial slip is beyond the scope of consideration.

### 2.2. Temperature Field

As temperature not only induces internal forces and deformation in the FML plate but also causes degradation of mechanical properties in constituent materials, the temperature field is first determined using heat conduction theory. According to the basic equations of the heat conduction theory [26], the heat conduction equation for the *i*-th lamina is given by

(1) $$k_{x}^{i}\frac{\partial^{2}\Delta T_{i}(x,y,z)}{\partial x^{2}}+k_{y}^{i}\frac{\partial^{2}\Delta T_{i}(x,y,z)}{\partial y^{2}}+k_{z}^{i}\frac{\partial^{2}\Delta T_{i}(x,y,z)}{\partial z^{2}}=0,$$

where $\Delta T_{i}$ represents the relative temperature with respect to the ambient temperature $T_{0}$, $k_{x}^{i}$, $k_{y}^{i}$, and $k_{z}^{i}$ represent the thermal conductivity coefficients in the *x*-, *y*-, and *z*-directions, respectively. The temperature field and the *z*-direction heat flux, denoted by $Q_{i}^{z}$, satisfy Fourier’s law of heat conduction:

(2) $$Q_{z}^{i}(x,y,z)=-k_{z}^{i}\frac{\partial \Delta T_{i}(x,y,z)}{\partial z}.$$

Equation (1), being a partial differential equation, is difficult to solve directly. Here, the temperature field is expanded into a Fourier series along the *x* and *y* directions [27]:

(3) $$\Delta T_{i}(x,y,z)=\sum_{m=1}^{\infty }\sum_{n=1}^{\infty }T_{mn}^{i}(z)\sin(\alpha_{m}x)\sin(\beta_{n}y),$$

where $\alpha_{m}=m\pi /a$, $\beta_{n}=n\pi /b$. Substituting the expanded expressions into Equation (1), one has

(4) $$\frac{d^{2}T_{mn}^{i}(z)}{dz^{2}}-{\left(p_{mn}^{i}\right)}^{2}t_{mn}^{i}(z)=0,$$

where $p_{mn}^{i}=\sqrt{k_{x}^{i}/k_{z}^{i}\alpha_{m}^{2}+k_{y}^{i}/k_{z}^{i}\beta_{n}^{2}}$. Solving the above equation leads to the general solutions of the temperature field and the heat flux:

$$\Delta T_{i}(x,y,z)=\sum_{m=1}^{\infty }\sum_{n=1}^{\infty }\left[\cosh(p_{mn}z)E_{mn}^{i}+\sinh(p_{mn}z)F_{mn}^{i}]\sin(\alpha_{m}x)\sin(\beta_{n}y\right),$$

(5) $$Q_{z}^{i}(x,y,z)=\sum_{m=1}^{\infty }\sum_{n=1}^{\infty }-k_{z}p_{mn}[\sinh(p_{mn}z)E_{mn}^{i}+\cosh(p_{mn}z)F_{mn}^{i}]\sin(\alpha_{m}x)\sin(\beta_{n}y),$$

where $E_{mn}^{i}$ and $F_{mn}^{i}$ are two undetermined coefficients.

Then, the general solutions for both the temperature field and the heat flux can be expressed compactly in matrix form as

(6) $$\Omega_{mn}^{i}(z)=B_{mn}^{i}(z)A_{mn}^{i},$$

where

$$\Omega_{mn}^{i}=\left[\begin{array}{c} \Delta T_{i}\\ Q_{z}^{i} \end{array}\right],\;B_{mn}^{i}=\left[\begin{array}{cc} \cosh(p_{mn}z) & \sinh(p_{mn}z)\\ -k_{z}p_{mn}\sinh(p_{mn}z) & -k_{z}p_{mn}\cosh(p_{mn}z) \end{array}\right],\;A_{mn}^{i}=\left[\begin{array}{c} F_{mn}^{i}\\ E_{mn}^{i} \end{array}\right].$$

By substituting the *z*-coordinates of the upper and lower surfaces of the *i*-th lamina into Equation (6) and eliminating $A_{mn}^{i}$, one obtains

$$\Omega_{mn}^{i}(z)=B_{mn}^{i}(z)B_{mn}^{i}{\left(d_{i-1}\right)}^{-1}\Omega_{mn}^{i}(d_{i-1}),$$

(7) $$\Omega_{mn}^{i}(d_{i})=B_{mn}^{i}(d_{i})B_{mn}^{i}{\left(d_{i-1}\right)}^{-1}\Omega_{mn}^{i}(d_{i-1}),$$

where $d_{i}=\sum_{j=1}^{i}h_{j}$. The continuity condition of temperature and heat flux in the *z*-direction at the interfaces is given by

(8) $$\Omega_{mn}^{i}(d_{i-1})=\Omega_{mn}^{i-1}(d_{i-1}),\;i\;=\;1,\;2\;...\;p\;-\;1.$$

By iteratively applying Equations (7) and (8), a direct relationship in terms of temperature and heat flux between the *i*-th lamina and the first lamina can be established:

(9) $$\Omega_{mn}^{i}(z)=B_{mn}^{i}(z)B_{mn}^{i}{\left(d_{i-1}\right)}^{-1}\prod_{j=i-1}^{1}\left[B_{mn}^{j}(d_{j})B_{mn}^{j}{\left(d_{j-1}\right)}^{-1}\right]\Omega_{mn}^{1}(0).$$

Setting *i* = *p* and *z* = *H* in Equation (9) provides the relationship between the top and bottom surfaces of the FML plate:

(10) $$\Omega_{mn}^{p}(H)=\prod_{j=p}^{1}\left[B_{mn}^{j}(d_{j})B_{mn}^{j}{\left(d_{j-1}\right)}^{-1}\right]\Omega_{mn}^{1}(0).$$

By substituting the surficial known temperatures into Equation (5), one obtains

(11) $$T_{mn}^{1}=E_{mn}^{1},\;T_{mn}^{p}=\cosh(p_{mn}H)E_{mn}^{p}+\sinh(p_{mn}H)F_{mn}^{p},$$

where

$$\left[\begin{array}{c} T_{mn}^{1}\\ T_{mn}^{p} \end{array}\right]=\frac{4}{ab}\int_{0}^{a}\int_{0}^{b}\left[\begin{array}{c} T_{1}\\ T_{2} \end{array}\right]\sin(\alpha_{m}x)\sin(\beta_{n}y)dxdy.$$

Substituting Equation (10) into Equation (6) yields

(12) $$A_{mn}^{p}=B_{mn}^{p}{\left(H\right)}^{-1}\left\{\prod_{j=p}^{1}\left[B_{mn}^{j}(d_{j})B_{mn}^{j}{\left(d_{j-1}\right)}^{-1}\right]\right\}B_{mn}^{1}(0)A_{mn}^{1}.$$

Then, by combining Equations (11) and (12), the following can be obtained:

(13) $$F_{mn}^{1}=\frac{T_{mn}^{p}-[\cosh(p_{mn}h_{p})C_{11}^{mn}+\sinh(p_{mn}h_{p})C_{21}^{mn}]T_{mn}^{1}}{\cosh(p_{mn}h_{p})C_{12}^{mn}+\sinh(p_{mn}h_{p})C_{22}^{mn}}$$

where

$$\left[\begin{array}{cc} C_{11}^{mn} & C_{12}^{mn}\\ C_{21}^{mn} & C_{22}^{mn} \end{array}\right]=B_{mn}^{p}{\left(H\right)}^{-1}\left\{\prod_{i=p}^{1}\left[B_{mn}^{i}(d_{i})B_{mn}^{i}{\left(d_{i-1}\right)}^{-1}\right]\right\}B_{mn}^{1}(0)$$

By substituting the obtained values of $E_{mn}^{1}$ and $F_{mn}^{1}$ into Equation (6), the result is $\Omega_{m}^{1}(0)$. Substituting $\Omega_{m}^{1}(0)$ into Equation (9) yields the analytical solution of the temperature field of the *i*-th lamina.

### 2.3. Stress and Displacement

To analyze the stress and displacement response of FML plate under coupled thermal and mechanical loading, the stress and displacement fields in the *i*-th lamina satisfy the governing equations of the three-dimensional thermoelasticity theory [28]. This theory’s fundamental equations focus on stresses, strains, and displacements, rather than bending moments or axial forces. In the FML plate, the FRP and the metal are orthotropic and isotropic materials, respectively. The constitutive relation for the *i*-th lamina is given by

(14) $$\left[\begin{array}{c} \sigma_{x}^{i}\\ \sigma_{y}^{i}\\ \sigma_{z}^{i}\\ \tau_{yz}^{i}\\ \tau_{xz}^{i}\\ \tau_{xy}^{i} \end{array}\right]=\left[\begin{array}{cccccc} c_{11}^{i} & c_{12}^{i} & c_{13}^{i} & 0 & 0 & 0\\ c_{12}^{i} & c_{22}^{i} & c_{23}^{i} & 0 & 0 & 0\\ c_{13}^{i} & c_{23}^{i} & c_{33}^{i} & 0 & 0 & 0\\ 0 & 0 & 0 & c_{44}^{i} & 0 & 0\\ 0 & 0 & 0 & 0 & c_{55}^{i} & 0\\ 0 & 0 & 0 & 0 & 0 & c_{66}^{i} \end{array}\right]\left(\left[\begin{array}{c} \varepsilon_{x}^{i}\\ \varepsilon_{y}^{i}\\ \varepsilon_{z}^{i}\\ \gamma_{yz}^{i}\\ \gamma_{xz}^{i}\\ \gamma_{xy}^{i} \end{array}\right]-\left[\begin{array}{c} \alpha_{x}^{i}\\ \alpha_{y}^{i}\\ \alpha_{z}^{i}\\ 0\\ 0\\ 0 \end{array}\right]\Delta T_{i}\right),$$

where $\sigma_{x}^{i}$, $\sigma_{y}^{i}$ and $\sigma_{z}^{i}$ denote the *x*-, *y*-, and *z*-direction normal stresses, respectively, while $\varepsilon_{x}^{i}$, $\varepsilon_{x}^{i}$ and $\varepsilon_{x}^{i}$ are the corresponding normal strains; $\tau_{yz}^{i}$, $\tau_{xz}^{i}$ and $\tau_{xy}^{i}$ denote the *yz*-, *xz*-, and *xy*-plane shear stresses, respectively, while $\gamma_{yz}^{i}$, $\gamma_{xz}^{i}$ and $\gamma_{xy}^{i}$ are the corresponding shear strains; $\alpha_{x}^{i}$, $\alpha_{y}^{i}$ and $\alpha_{z}^{i}$ represents the *x*-, *y*-, and *z*-direction thermal expansion coefficient, respectively; $c_{**}^{i}$ represents the stiffness coefficient, and for orthotropic materials, it can be expressed as

$$c_{11}^{i}=\frac{1-\mu_{23}^{i}\mu_{32}^{i}}{E_{2}^{i}E_{3}^{i}\Delta^{i}}\phi_{i},\;c_{12}^{i}=\frac{\mu_{12}^{i}+\mu_{13}^{i}\mu_{32}^{i}}{E_{1}^{i}E_{3}^{i}\Delta^{i}}\phi_{i},\;c_{13}^{i}=\frac{\mu_{13}^{i}+\mu_{12}^{i}\mu_{23}^{i}}{E_{1}^{i}E_{2}^{i}\Delta^{i}}\phi_{i},$$

$$c_{22}^{i}=\frac{1-\mu_{13}^{i}\mu_{31}^{i}}{E_{1}^{i}E_{3}^{i}\Delta^{i}}\phi_{i},\;c_{23}^{i}=\frac{\mu_{23}^{i}+\mu_{21}^{i}\mu_{13}^{i}}{E_{1}^{i}E_{2}^{i}\Delta^{i}}\phi_{i},\;c_{33}^{i}=\frac{1-\mu_{12}^{i}\mu_{21}^{i}}{E_{1}^{i}E_{2}^{i}\Delta^{i}}\phi_{i},$$

$$c_{44}^{i}=G_{23}^{i}\phi_{i},\;c_{55}^{i}=G_{13}^{i}\phi_{i},\;c_{66}^{i}=G_{12}^{i}\phi_{i},$$

(15) $$\Delta^{i}=\left|\begin{array}{ccc} \frac{1}{E_{1}^{i}} & -\frac{\mu_{21}^{i}}{E_{2}^{i}} & -\frac{\mu_{31}^{i}}{E_{3}^{i}}\\ -\frac{\mu_{12}^{i}}{E_{1}^{i}} & \frac{1}{E_{2}^{i}} & -\frac{\mu_{32}^{i}}{E_{3}^{i}}\\ -\frac{\mu_{13}^{i}}{E_{1}^{i}} & -\frac{\mu_{23}^{i}}{E_{2}^{i}} & \frac{1}{E_{3}^{i}} \end{array}\right|,$$

where ϕ denotes the temperature influence factor, *E* and *G* denote the elastic and shear modulus, respectively, and μ is the Poisson’s ratio. Subscripts 1, 2, and 3 refer to the principal material directions aligned with the global *x*-, *y*-, and *z*-axes. Isotropic materials can be recognized as a special case of anisotropic materials, with *E* = *E*_1_ = *E*_2_ = *E*_3_, *G* = *G*_1_ = *G*_2_ = *G*_3_, *µ* = *µ*_12_ = *µ*_13_ = *µ*_23_, *E* = 2(1 + *µ*)*G*. In addition, the strain–displacement relations and equilibrium equations are given by

(16) $$\frac{\partial \sigma_{x}^{i}}{\partial x}+\frac{\partial \tau_{xy}^{i}}{\partial y}+\frac{\partial \tau_{xz}^{i}}{\partial z}=0,\;\frac{\partial \sigma_{y}^{i}}{\partial y}+\frac{\partial \tau_{xy}^{i}}{\partial x}+\frac{\partial \tau_{yz}^{i}}{\partial z}=0,\;\frac{\partial \sigma_{z}^{i}}{\partial z}+\frac{\partial \tau_{xz}^{i}}{\partial x}+\frac{\partial \tau_{yz}^{i}}{\partial y}=0,$$

(17) $$\varepsilon_{x}^{i}=\frac{\partial u^{i}}{\partial x},\;\varepsilon_{y}^{i}=\frac{\partial v^{i}}{\partial y},\;\varepsilon_{z}^{i}=\frac{\partial w^{i}}{\partial z},\;\gamma_{yz}^{i}=\frac{\partial v^{i}}{\partial z}+\frac{\partial w^{i}}{\partial y},\;\gamma_{xz}^{i}=\frac{\partial u^{i}}{\partial z}+\frac{\partial w^{i}}{\partial x},\;\gamma_{xy}^{i}=\frac{\partial u^{i}}{\partial y}+\frac{\partial v^{i}}{\partial x},$$

where $u^{i}$, $v^{i}$, and $w^{i}$ correspond to the displacements in the *x*-, *y*-, and *z*-directions.

The simply supported boundary conditions applied to the FML plate are

$$\sigma_{x}^{i}=v^{i}=w^{i}=0,\;\mathrm{at}\;x=0,a,$$

(18) $$\sigma_{y}^{i}=u^{i}=w^{i}=0,\;\mathrm{at}\;y=0,b.$$

Given the above boundary conditions, the stresses and displacements are represented using a Fourier series expansion:

(19) $$\left[\begin{array}{l} u^{i}\\ v^{i}\\ w^{i}\\ \sigma_{z}^{i}\\ \tau_{xz}^{i}\\ \tau_{yz}^{i} \end{array}\right]=\sum_{m=1}^{\infty }\sum_{n=1}^{\infty }\left[\begin{array}{l} u_{mn}^{i}\cos(\alpha_{m}x)\sin(\beta_{n}y)\\ v_{mn}^{i}\sin(\alpha_{m}x)\cos(\beta_{n}y)\\ w_{mn}^{i}\sin(\alpha_{m}x)\sin(\beta_{n}y)\\ \sigma_{z,mn}^{i}\sin(\alpha_{m}x)\sin(\beta_{n}y)\\ \tau_{xz,mn}^{i}\cos(\alpha_{m}x)\sin(\beta_{n}y)\\ \tau_{yz,mn}^{i}\sin(\alpha_{m}x)\cos(\beta_{n}y) \end{array}\right],\;\left[\begin{array}{c} \sigma_{x}^{i}\\ \sigma_{y}^{i}\\ \tau_{xy}^{i} \end{array}\right]=\sum_{m=1}^{\infty }\sum_{n=1}^{\infty }\left[\begin{array}{c} \sigma_{x,mn}^{i}\sin(\alpha_{m}x)\sin(\beta_{n}y)\\ \sigma_{y,mn}^{i}\sin(\alpha_{m}x)\sin(\beta_{n}y)\\ \tau_{xy,mn}^{i}\cos(\alpha_{m}x)\cos(\beta_{n}y) \end{array}\right].$$

Based on the derivation procedure of the state-space method [29], and by substituting the above Fourier series expansions into Equations (14), (16) and (17), the state-space equation can be obtained as follows:

(20) $$\frac{\partial }{\partial y}[X_{mn}^{i}(z)]=D_{mn}^{i}X_{mn}^{i}(z)+T_{mn}^{i}(z),$$

where

$$D_{mn}^{i}=\left[\begin{array}{cccccc} 0 & 0 & -\alpha_{m} & 0 & \frac{1}{c_{55}^{i}} & 0\\ 0 & 0 & -\beta_{n} & 0 & 0 & \frac{1}{c_{44}^{i}}\\ \frac{\alpha_{m}c_{13}^{i}}{c_{33}^{i}} & \frac{\beta_{n}c_{23}^{i}}{c_{33}^{i}} & 0 & \frac{1}{c_{33}^{i}} & 0 & 0\\ 0 & 0 & 0 & 0 & \alpha_{m} & \beta_{n}\\ f_{1}^{i}\alpha_{m}^{2}+c_{66}^{i}\beta_{n}^{2} & (f_{2}^{i}+c_{66}^{i})\alpha_{m}\beta_{n} & 0 & -\frac{\alpha_{m}c_{13}^{i}}{c_{33}^{i}} & 0 & 0\\ (f_{2}^{i}+c_{66}^{i})\alpha_{m}\beta_{n} & f_{3}^{i}\beta_{n}^{2}+c_{66}^{i}\alpha_{m}^{2} & 0 & -\frac{\beta_{n}c_{23}^{i}}{c_{33}^{i}} & 0 & 0 \end{array}\right],\;X_{mn}^{i}(z)=\left[\begin{array}{c} u_{mn}^{i}\\ v_{mn}^{i}\\ w_{mn}^{i}\\ \sigma_{z,mn}^{i}\\ \tau_{xz,mn}^{i}\\ \tau_{yz,mn}^{i} \end{array}\right],\;T_{mn}^{i}(z)=\left[\begin{array}{c} 0\\ 0\\ \frac{c_{13}^{i}\alpha_{x}^{i}+c_{23}^{i}\alpha_{y}^{i}+c_{33}^{i}\alpha_{z}^{i}}{c_{33}^{i}}T_{mn}^{i}\\ 0\\ \alpha_{m}(f_{1}^{i}\alpha_{x}^{i}+f_{2}^{i}\alpha_{y}^{i})T_{mn}^{i}\\ \beta_{n}(f_{2}^{i}\alpha_{x}^{i}+f_{3}^{i}\alpha_{y}^{i})T_{mn}^{i} \end{array}\right],\;\left[\begin{array}{c} f_{1}^{i}\\ f_{2}^{i}\\ f_{3}^{i} \end{array}\right]=\frac{1}{c_{33}^{i}}\left[\begin{array}{c} c_{11}^{i}c_{33}^{i}-{\left(c_{13}^{i}\right)}^{2}\\ c_{12}^{i}c_{33}^{i}-c_{13}^{i}c_{23}^{i}\\ c_{22}^{i}c_{33}^{i}-{\left(c_{23}^{i}\right)}^{2} \end{array}\right].$$

By solving Equation (20), the general solutions for the out-of-plane stress and displacement components can be obtained as

(21) $$X_{mn}^{i}(z)=e^{D_{mn}^{i}(z-d_{i-1})}X_{mn}^{i}(d_{i-1})+K_{i}(z),$$

where

$$K_{i}(z)=\int_{d_{i-1}}^{z}e^{D_{mn}^{i}(z-\zeta )}T_{mn}^{i}(\zeta )d\zeta .$$

According the governing equations, the in-plane quantities are related to the out-of-plane ones as follows:

(22) $$\left[\begin{array}{c} \sigma_{x,mn}^{i}\\ \sigma_{y,mn}^{i}\\ \tau_{xy,mn}^{i} \end{array}\right]=\left[\begin{array}{ccc} -f_{1}^{i}\alpha_{m} & -f_{2}^{i}\beta_{n} & \frac{c_{13}^{i}}{c_{33}^{i}}\\ -f_{2}^{i}\alpha_{m} & -f_{3}^{i}\beta_{n} & \frac{c_{23}^{i}}{c_{33}^{i}}\\ c_{66}^{i}\beta_{n} & c_{66}^{i}\alpha_{m} & 0 \end{array}\right]\left[\begin{array}{c} u_{mn}^{i}\\ v_{mn}^{i}\\ \sigma_{z,mn}^{i} \end{array}\right]+\left[\begin{array}{c} -f_{1}^{i}\alpha_{x}^{i}-f_{2}^{i}\alpha_{y}^{i}\\ -f_{2}^{i}\alpha_{x}^{i}-f_{3}^{i}\alpha_{y}^{i}\\ 0 \end{array}\right]T_{mn}^{i}.$$

Given the well-bonded interfacial conditions, the out-of-plane stresses and displacements are continuous across adjacent laminae, as follows:

(23) $$X_{mn}^{i}(d_{i-1})=X_{mn}^{i-1}(d_{i-1}),\;i=1,2\ldots p-1.$$

By letting $z=d_{i}$ in Equation (21), one has

(24) $$X_{mn}^{i}(d_{i})=e^{D_{mn}^{i}(h_{i})}X_{mn}^{i}(d_{i-1})+K_{i}(d_{i}).$$

Analogous to the process of solving temperature fields, the relationship of stresses and displacements in different laminae can also be established by using the transfer matrix method. Repeated application of Equations (23) and (24) yields a direct relationship between the *i*-th and the first lamina:

(25) $$X_{mn}^{i}(z)=L_{mn}^{i}(z)X_{mn}^{1}(0)+N_{mn}^{i}(z),$$

where

$$\begin{array}{c} L_{mn}^{i}(z)=e^{D_{m}^{i}(z-d_{i-1})}\prod_{k=i-1}^{1}e^{D_{m}^{k}h_{k}},\\ N_{mn}^{i}(z)=e^{D_{m}^{i}(z-d_{i-1})}\sum_{k=1}^{i-2}\left\{\left[\prod_{j=i-1}^{k+1}e^{D_{m}^{j}h_{j}}\right]K_{k}(h_{k})\right\}+e^{D_{m}^{i}(z-d_{i-1})}K_{i-1}(h_{i-1})+K_{i}(z). \end{array}$$

The next step is to determine $X_{mn}^{1}(0)$ in Equation (25), in which the surficial load condition is known as

$$\sigma_{z}^{1}=\tau_{xz}^{1}=\tau_{yz}^{1}=0,\;\mathrm{at}\;z=0,$$

(26) $$\sigma_{z}^{p}=-q(x,y),\;\tau_{xz}^{p}=\tau_{yz}^{p}=0,\;\mathrm{at}\;z=H.$$

Substituting Equation (26) into Equation (25) and letting *i* = *p*, z=H, lead to

(27) $$\left[\begin{array}{c} u_{mn}^{p}(H)\\ v_{mn}^{p}(H)\\ w_{mn}^{p}(H)\\ -q_{mn}\\ 0\\ 0 \end{array}\right]=L_{mn}^{p}(H)\left[\begin{array}{c} u_{mn}^{1}(0)\\ v_{mn}^{1}(0)\\ w_{mn}^{1}(0)\\ 0\\ 0\\ 0 \end{array}\right]+N_{mn}^{p}(H),$$

where

$$q_{mn}=\frac{4}{ab}\int_{0}^{a}\int_{0}^{b}F(x,y)\sin(\alpha_{m}x)\sin(\beta_{n}y)dxdy.$$

The unknown displacements in $X_{mn}^{1}(0)$ can be determined by solving Equation (27) as given below:

(28) $$\left[\begin{array}{c} u_{mn}^{1}(0)\\ v_{mn}^{1}(0)\\ w_{mn}^{1}(0) \end{array}\right]={\left(\Phi_{mn}^{21}\right)}^{-1}\left\{\left[\begin{array}{c} -q_{mn}\\ 0\\ 0 \end{array}\right]-\Psi_{mn}^{2}\right\},$$

where

$$\left[\begin{array}{cc} \Phi_{11} & \Phi_{12}\\ \Phi_{21} & \Phi_{22} \end{array}\right]=L_{mn}^{p}(H),\;\left[\begin{array}{c} \Psi_{mn}^{1}\\ \Psi_{mn}^{2} \end{array}\right]=N_{mn}^{p}(H).$$

So far, $X_{mn}^{1}(0)$ is obtained, and substituting it back into Equation (25) yields the analytical solutions of stress and displacement fields of the FML plate under thermo-mechanical loads.

**Figure 2:**
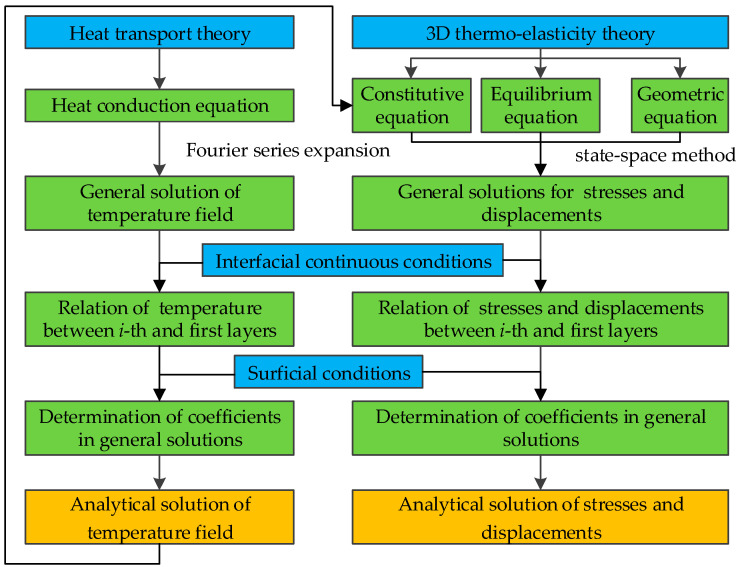
Flowchart of the present analytical model.

To improve clarity, Figure 2 illustrates the flowchart of the analytical model developed in the present study.

## 3. Results and Discussion

The FML plate composed of aluminum alloy (AA) and carbon fiber reinforced polymer (CFRP) represents a typical configuration of FML plates and is selected in this study as the object of analysis. According to experiment results [30,31], the material properties of AA and CFRP with *T*_0_ = 20 °C are listed in Table 1, and their modulus degradation factors are given by

$$\phi_{\mathrm{AA}}=0.5-0.5\tanh[0.0062(T-273)],$$

(29) $$\phi_{\mathrm{CFRP}}=1-\ln\left[\left(1-\frac{T+273}{431}\right)/\left(1-\frac{293}{431}\right)\right]/5\ln(1-\frac{293}{431}),$$

where *T* denotes actual temperature with $T=\Delta T+T_{0}$. The angle between the CFRP fiber direction and the *x*-axis is defined as *θ*.

### 3.1. Comparison and Decoupling Analyses

This section investigates stresses and displacements of FML plate under thermo-mechanical loads. Consider a two-layer FML plate composed of an AA top lamina and a CFRP bottom lamina with *θ* = 0. under a mechanical load *q*(*x*) = 0.03 N/mm^2^ in uniform temperature *T* = 120 °C, with geometric parameters taken as *a* = *b* = 150 mm, *h*_1_ = *h*_2_ = 0.6 mm. To study the thermo-mechanical coupling effect, four analysis conditions are defined in advance, including pure mechanical (PM) load, pure temperature (PT) environment, mechanical load but considering temperature-induced modulus degradation (MD), and thermo-mechanical (MT) loads, as specifically listed in Table 2.

**Figure 3:**
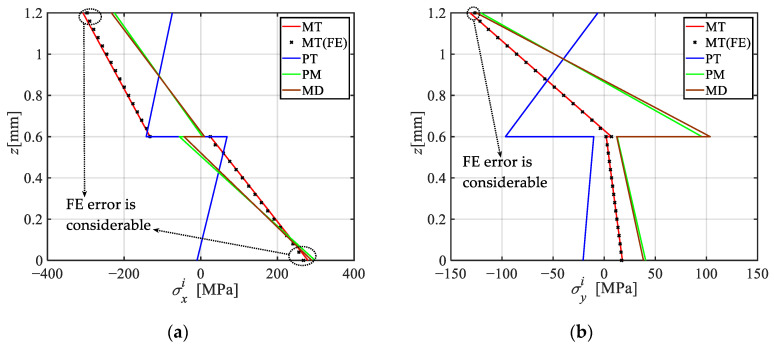

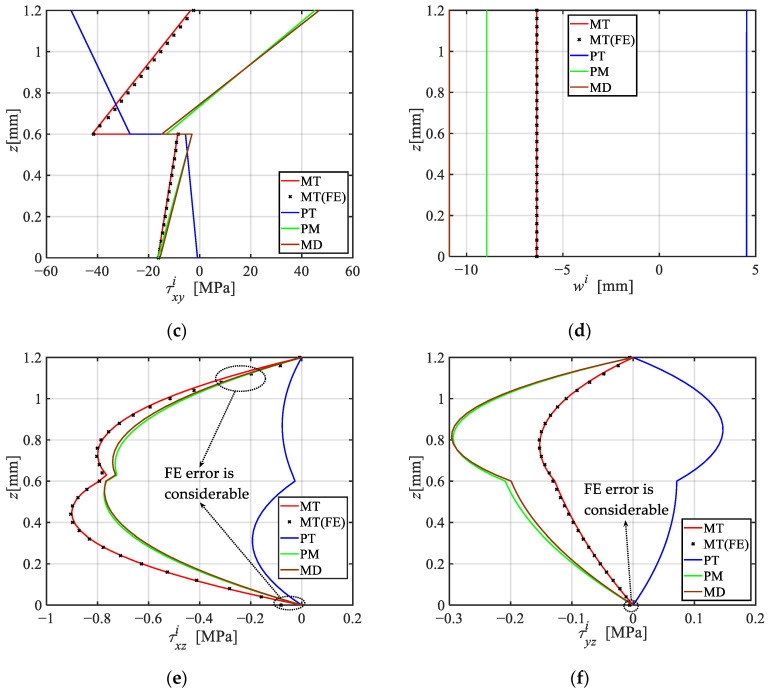
Distribution of stress and displacement along the thickness under the four analysis environments and comparison with FE results. (**a**) $\sigma_{x}^{i}$ at *x* = 0.5*a*, *y* = 0.5*b*; (**b**) $\sigma_{y}^{i}$ at *x* = 0.5*a*, *y* = 0.5*b*; (**c**) $\tau_{\mathrm{xy}}^{i}$ at *x* = 0.25*a*, *y* = 0.25*b*; (**d**) *w* at *x* = 0.5*a*, *y* = 0.5*b*; (**e**) $\tau_{\mathrm{xz}}^{i}$ at *x* = 0.25*a*, *y* = 0.25*b*; (**f**) $\tau_{\mathrm{yz}}^{i}$ at *x* = 0.25*a*, *y* = 0.25*b*.

Figure 3 illustrates the through-thickness distributions of stresses and displacements under four analysis conditions, alongside comparisons with finite element (FE) results obtained using commercial software ANSYS (version: 2023). In the FE model, thermal analysis of the FML plate begins with the SOLID-70 element to establish the temperature field. Subsequently, this temperature field and the surface load are applied to the FML plate using the SOLID-185 element to compute stresses and displacements, utilizing a total of 2,160,000 elements. It can be observed from Figure 3 that (i) overall, the FE results agree well with the present analytical solutions, with a relative error of less than 2%, except for considerable errors near the upper and lower surfaces, as indicated by the dashed frame. The occurrence of these errors is primarily attributed to ANSYS nodal extrapolation, with additional contributions from the element order and the integration strategy. (ii) Under PM loading, the maximum values of $\sigma_{x}^{i}$ and $\sigma_{y}^{i}$ are located at the top and bottom surfaces of the plate, indicating bending characteristics. Conversely, in the PT environment, these maximums occur at the interface, mainly attributable to differences in thermal expansion coefficients. (iii) the stress and displacement directions under the PT environment and the PM condition are generally opposite, primarily due to the significantly higher thermal expansion coefficient of AA in the *x*-direction compared to that of CFRP, while the plate bends downward under PM loading. The coupling of thermal and mechanical loading can, to some extent, mitigate the stresses and displacements in the plate. (iv) $\sigma_{x}^{i}$, $\sigma_{y}^{i}$, and $\tau_{xy}^{i}$ exhibit zigzag distributions with discontinuities at the interface, whereas $\tau_{xz}^{i}$ and $\tau_{yz}^{i}$ show multi-peak profiles. In both PM and PT conditions, the lamina with a higher elastic modulus exhibits greater stress values, steeper gradients in $\sigma_{x}^{i}$, $\sigma_{y}^{i}$, and $\tau_{xy}^{i}$, and more pronounced curvature in $\tau_{xz}^{i}$, $\tau_{yz}^{i}$. (v) Analysis of stress and displacement values reveals the inadequacy of the traditional superposition principle (TSP). Accordingly, a modified superposition principle (MSP) that incorporates temperature-induced modulus degradation is proposed, as follows:

TSP:PM+PT≠MT,

(30) MSP:MD+PT=MT.

Table 3 lists the maximum stresses and displacements calculated using TSP and MSP, respectively, together with the errors associated with TSP. As can be seen from Table 3, the errors in shear stresses are the smallest, below 1%; the errors in normal stresses are less than 5%; whereas the error in displacement *w* is the most significant, reaching 30.39%.

The present solution can also be applied to other laminated plates, such as cross-laminated timber (CLT) plates. To verify the present solution, it is compared with the experimental results for CLT plates reported in Ref. [33]. Two CLT plates made of Canadian black spruce were tested, including a three-layer plate (3300 mm long, 35/35/35 mm laminations) and a five-layer plate (4800 mm long, 35/25/35/25/35 mm laminations). For each type of CLT plate, three repeated tests were conducted, with all specimens subjected to bending along the major strength direction. Table 4 compares the present results with the experimental data from three repeated tests within the linear range. It can be found from Table 4 that the present results are close to the experimental results, with errors less than 10%.

### 3.2. Effect of Surficial Temperature Difference

**Figure 4:**
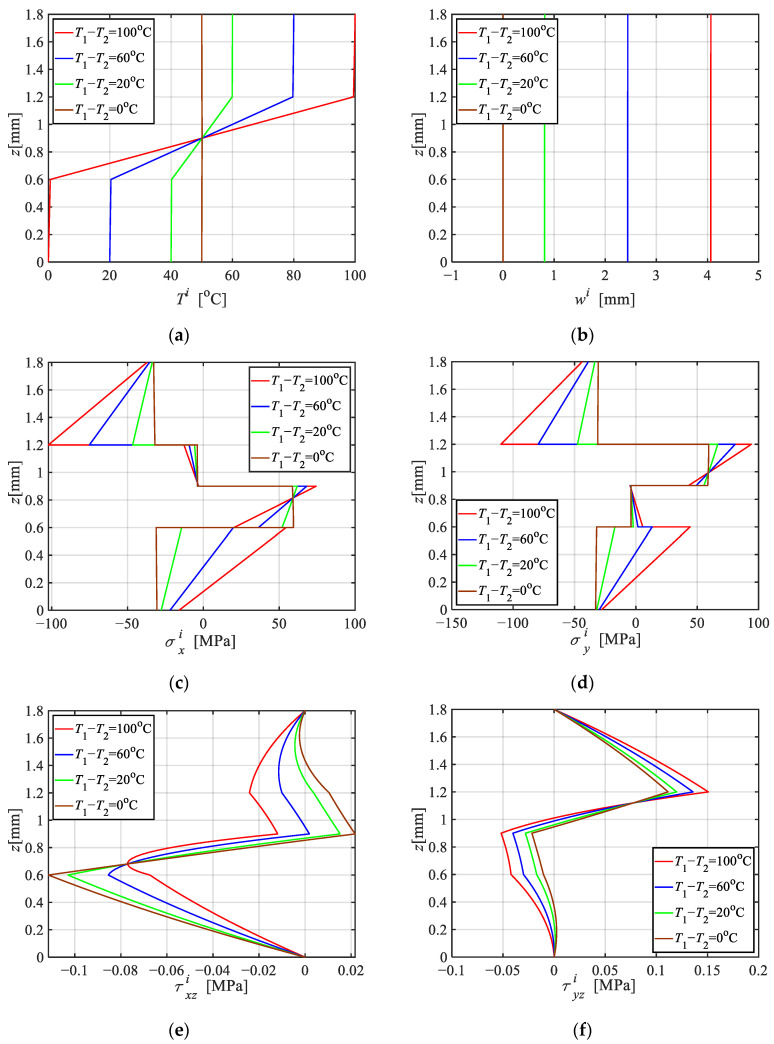
Distribution of stress and displacement along the thickness of the FML plate under the four temperature gradients. (**a**) $T^{i}$ at *x* = 0.5*a*, *y* = 0.5*b*; (**b**) *w* at *x* = 0.5*a*, *y* = 0.5*b*; (**c**) $\sigma_{x}^{i}$ at *x* = 0.5*a*, *y* = 0.5*b*; (**d**) $\sigma_{y}^{i}$ at *x* = 0.5*a*, *y* = 0.5*b*; (**e**) $\tau_{\mathrm{xz}}^{i}$ at *x* = 0.25*a*, *y* = 0.25*b*; (**f**) $\tau_{\mathrm{yz}}^{i}$ at *x* = 0.25*a*, *y* = 0.25*b*.

This section investigates the effect of temperature differences between the top and bottom surfaces on thermal stresses and displacements. Consider a four-layer FML plate, arranged from top to bottom as AA, CFRP with *θ* = 0, CFRP with *θ* = π/2, AA, with geometric sizes taken as *a* = *b* = 150 mm, *h*_1_ = *h*_4_ = 0.6 mm, *h*_2_ = *h*_3_ = 0.3 mm. Figure 4 illustrates the temperature, stress and displacement distributions along the thickness under four surficial temperature differences: *T*_1_ − *T*_2_ = 0, 20, 60, and 100 °C, respectively.

The following conclusions can be drawn from Figure 4: (i) the temperature gradient within the AA lamina is significantly smaller than that in the CFRP lamina, due to the much higher thermal conductivity of AA compared to CFRP. (ii) Due to the significantly higher thermal expansion coefficient of AA compared to CFRP in the fiber direction, thermal loading induces tensile stress in AA and compressive stress in CFRP. (iii) In a uniform temperature field, each lamina experiences a constant normal stress and a shear stress that varies linearly across its thickness. The distribution of $\sigma_{x}^{i}$, $\sigma_{y}^{i}$ across the laminae is approximately proportional to their respective elastic moduli. (iv) By contrast, a non-uniform temperature field leads to differential thermal expansion across the thickness, thereby exhibiting bending behavior. As a result, $\sigma_{x}^{i}$ and $\sigma_{y}^{i}$ become linearly distributed. With increasing temperature gradient, both the $\sigma_{x}^{i}$ and $\sigma_{y}^{i}$ slopes become more pronounced, driven by the bending effect.

### 3.3. Combined Effects of Temperature and Lamina Number

The combined effects of the lamina number and temperature magnitude on the thermal stresses and displacements are investigated in this section. Consider an FML plate consisting of *p* laminae formed by alternating laminae of AA and CFRP with *θ* = 0, starting with an AA lamina (*i* = 1), subjected to a uniform temperature *T*, with geometric sizes taken as *a* = 1200 mm, *b* = 1500 mm, and *h_i_* = 100/*p* mm.

**Figure 5:**
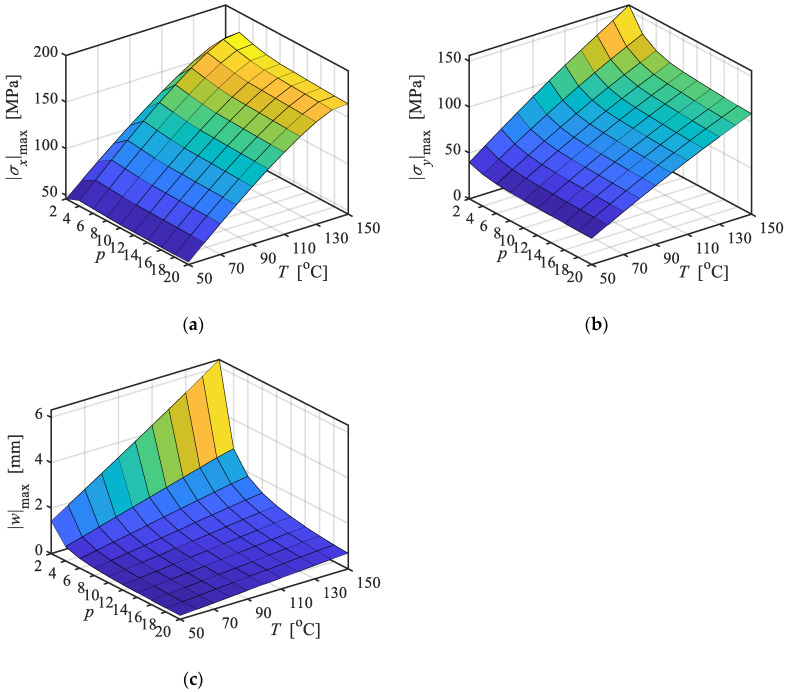
Variations in stresses and displacement of FML plate with temperatures and lamina number. (**a**) $|\sigma_{x}{|}_{\max}$ at *x* = 0.5*a*, *y* = 0.5*b*; (**b**) $|\sigma_{y}{|}_{\max}$ at *x* = 0.5*a*, *y* = 0.5*b*; (**c**) $|w{|}_{\max}$ at *x* = 0.5*a*, *y* = 0.5*b*.

Figure 5 shows the thermal stresses and displacements vary with *p* and *T*, in which *p* is even number. It can be found that (i) as the *T* rises, the maximum stresses shows nonlinear increase, with the growth rate gradually decreasing. This is due to the temperature-dependent degradation of the elastic modulus, as higher temperatures significantly reduce the modulus of the constituent materials. (ii) With increasing *p*, $|\sigma_{x}{|}_{\max}$ and $|\sigma_{y}{|}_{\max}$ tend to decrease and gradually stabilize, except at low *p* values. This behavior is primarily attributed to the more uniform distribution of the stresses across each lamina as *p* increases. (iii) For small *p* values, $|w{|}_{\max}$ decreases rapidly as *p* increases; for larger *p*, the rate of decrease slows down and $|w{|}_{\max}$ gradually approaches a stable value. This behavior is attributed to the asymmetric material distribution across the cross-section when *p* = 2, which causes the plate to arch downward under a uniform temperature field. As *p* increases, the degree of asymmetry gradually diminishes.

## 4. Conclusions

In order to investigate the thermo-mechanical bending behavior of FML plates, an exact analytical model based on 3D thermos-elasticity theory is developed in this study. The main findings are summarized as follows:

1. The finite element results generally agree well with the present analytical solutions, with relative errors below 2%, except in regions adjacent to the top and bottom surfaces. In addition, the present solution agrees well with the experimental values for the laminated plate within the linear range, with errors below 10%.
2. The traditional superposition principle proves inaccurate in predicting the thermo-mechanical bending behavior of FML plates, with maximum errors reaching up to 30.39%. To address this, a modified superposition principle incorporating temperature-induced modulus reduction is proposed.
3. Under a uniform temperature field, normal stress remains constant and shear stress varies linearly across each lamina. A non-uniform temperature field causes differential thermal expansion, leading to bending deformation; the normal stress slope increases with the temperature gradient, amplifying the bending effect.
4. The combined effect of temperature and lamina number shows that increasing temperature leads to nonlinear stress growth due to modulus degradation, while increasing the number of laminae contributes to stress distribution homogenization. For small lamina counts, displacements decrease rapidly with increasing lamina number, whereas beyond a certain threshold, the benefits of additional laminae diminish and the response approaches a stable value.

For future work, the present theoretical model will be expanded to incorporate the viscoelastic properties of FML plates under thermo-mechanical loads.

## Figures and Tables

**Table 1:** Material properties of constituent materials [32].

Material Type	Values of Material Property
CFRP	$E_{11}$ $=\;153,\;E_{22}$ $=\;10.3,\;E_{33}$ $=\;10.3,\;G_{12}$ $=\;6,\;G_{13}$ = 6, $G_{23}$ $=\;3.7,\;\mu_{12}$ $=\;0.3,\;\mu_{13}$ $=\;0.3,\;\mu_{23}$ = 0.4 $\alpha_{1}$ $=\;-0.5\times 10^{-6},\;\alpha_{2}$ $=\;30\;\times \;10^{-6},\;\alpha_{3}$ = 30 × 10^−6^, $k_{1}$ $=\;8.5,\;k_{2}$ $=\;0.8,\;k_{3}$ = 0.8
AA	E=70, G=26.3, μ = 0.33 α = 23.6 × 10^−6^, *k* = 121

Note: 1. the units of modulus and k are [GPa] and [W/m °C], respectively; 2. the material principal directions 1 and 2 are interchanged accordingly, when the fiber direction is perpendicular to the global *x*-axis.

**Table 2:** Definitions of the four analysis conditions.

Conditions	Load Effect	Thermal Stresses and Deformation	Temperature-Induced Modulus Degradation
PM	√		
PT		√	√
MD	√		√
MT	√	√	√

**Table 3:** Errors of maximum stresses and displacements between TSP and MSP.

Conditions	$|\sigma_{x}^{i}{|}_{\max}$ (MPa)	$|\sigma_{y}^{i}{|}_{\max}$ (MPa)	$|\tau_{xy}^{i}{|}_{\max}$ (MPa)	$|\tau_{xz}^{i}{|}_{\max}$ (MPa)	$|\tau_{yz}^{i}{|}_{\max}$ (MPa)	$|w^{i}{|}_{\max}$ (mm)
TSP (PM + PT)	299.4	126.83	40.33	0.9094	0.1527	4.430
MSP (MT)	307.2	131.2	42.10	0.9012	0.1516	6.364
errors	2.54%	3.33%	4.20%	0.91%	0.73%	30.39%

**Table 4:** Comparison between the present results and experimental results from three repeated tests.

Solution	Displacement in Linear Rage (mm)	
	Three-Layer	Five-Layer
Present	62.49	83.77
Test 1	59.5	85.76
Test 2	69.47	87.19
Test 3	75.12	104
Test average	68.03	92.32
Errors	8.14%	9.26%

## Data Availability

The original contributions presented in this study are included in the article. Further inquiries can be directed to the corresponding author.
